# Alterations of Subchondral Bone Progenitor Cells in Human Knee and Hip Osteoarthritis Lead to a Bone Sclerosis Phenotype

**DOI:** 10.3390/ijms19020475

**Published:** 2018-02-06

**Authors:** Daniel Bianco, Atanas Todorov, Tomislav Čengić, Geert Pagenstert, Stefan Schären, Cordula Netzer, Thomas Hügle, Jeroen Geurts

**Affiliations:** 1 Orthopaedic Department, University Hospital of Basel, 4031 Basel, Switzerland; danbiancocot@gmail.com (D.B.); cengict@me.com (T.Č.); pagenstert@praxisclarahof.ch (G.P.); 2 Tissue Engineering, Department of Biomedicine, University Hospital of Basel, 4031 Basel, Switzerland; atanastodo@gmail.com; 3 Department of Traumatology, University Hospital Centre Sestre Milosrdnice, 10000 Zagreb, Croatia; 4 Department of Spine Surgery, University Hospital of Basel, 4031 Basel, Switzerland; Stefan.Schaeren@usb.ch (S.S.); Cordula.Netzer@usb.ch (C.N.); 5 Department of Rheumatology, University Hospital Lausanne (CHUV), 1005 Lausanne, Switzerland; Thomas.Hugle@chuv.ch; 6 Department of Biomedical Engineering, University Hospital of Basel, 4123 Allschwil, Switzerland

**Keywords:** osteoarthritis, subchondral bone, osteoprogenitors, osteogenic differentiation, ectopic bone formation, clonogenicity, computed tomography

## Abstract

Subchondral bone tissue plays a key role in the initiation and progression of human and experimental osteoarthritis and has received considerable interest as a treatment target. Elevated bone turnover and remodeling leads to subchondral bone sclerosis that is characterized by an increase in bone material that is less mineralized. The aim of this study was to investigate whether perturbations in subchondral bone-resident progenitor cells might play a role in aberrant bone formation in osteoarthritis. Colony formation assays indicated similar clonogenicity of progenitor cells from non-sclerotic and sclerotic subchondral trabecular bone tissues of osteoarthritic knee and hip joints compared with controls from iliac crest bone. However, the osteogenic potential at the clonal level was approximately two-fold higher in osteoarthritis than controls. An osteogenic differentiation assay indicated an efficient induction of alkaline phosphatase activity but blunted in vitro matrix mineralization irrespective of the presence of sclerosis. Micro-computed tomography and histology demonstrated the formation of de novo calcified tissues by osteoblast-like cells in an ectopic implantation model. The expression of bone sialoprotein, a marker for osteoblast maturation and mineralization, was significantly less in sclerotic progenitor cells. Perturbation of resident progenitor cell function is associated with subchondral bone sclerosis and may be a treatment target for osteoarthritis.

## 1. Introduction

Osteoarthritis (OA) is a chronic disorder involving the movable joints. It is characterized by cell stress and extracellular matrix degradation, initiated by micro- and macro-injury, that activates maladaptive repair responses including pro-inflammatory pathways of innate immunity. The disease manifests first as a molecular derangement (i.e., abnormal joint tissue metabolism) followed by anatomic and physiologic derangements that culminate in illness [1]. The prevalence of OA increases steeply with age and poses a major socioeconomic burden on society. OA is characterized by progressive loss of articular cartilage, elevated turnover and remodeling of subjacent subchondral bone and marrow tissues, and synovial tissue inflammation [2]. While the exact pathomechanisms remain largely elusive, pharmacological targeting of subchondral bone turnover and remodeling has demonstrated disease-modifying effects in human and experimental knee OA [3,4,5,6]. Nevertheless, an effective non-surgical therapy is still lacking and, therefore, partial or total joint replacement is the routine treatment strategy for end-stage disease.

Subchondral bone sclerosis, defined as an increase in bone mass and density, is a common radiographical feature of advanced disease stages. A marked characteristic of subchondral bone sclerosis is the formation of new bone tissue that has lower mineralization [7,8,9]. As a consequence, the strength and stiffness of the bone tissue are altered, leading to increased brittleness of the sclerotic subchondral bone [7]. Computational models have shown that less tissue mineralization drives the increase in bone mass in OA joint remodeling [10]. The molecular and cellular mechanisms underpinning subchondral bone sclerosis and hypomineralization are incompletely understood. Phenotypical characterizations of primary outgrowth osteoblasts from non-sclerotic and sclerotic subchondral bone tissue of OA patients have identified several molecular alterations that are associated with less in vitro matrix mineralization in the latter [11,12,13,14]. Alkaline phosphatase (ALP) levels, reflecting the biosynthetic activity of osteoblasts, were found to be specifically elevated in outgrowth cells from sclerotic bone [11,14]. Aberrant and increased production of type I collagen, the major organic component of bone matrix, due to altered transforming growth factor-β (TGF-β) and WNT/β-catenin signaling has been identified as the major molecular mechanism underlying decreased matrix mineralization in sclerotic osteoblasts [12,13]. While these studies have provided valuable insight into the molecular derangements of subchondral bone-resident cells, it is unknown how and when the genotypical and phenotypical changes in osteoblasts are acquired.

Recent studies have shown enhanced recruitment of osteoprogenitor/multipotential stromal cells into the marrow tissue of remodeling subchondral bone in experimental and human OA [5,15]. Osteogenic differentiation of recruited progenitor cells resulted in the formation of osteoid islets in marrow tissues, giving rise to subchondral bone sclerosis [5]. In vitro characterization of clonogenic progenitor cells isolated from marrow tissues in hip OA has revealed molecular alterations in cells derived from subchondral bone tissues, displaying elevated turnover and remodeling [15]. Specifically, calcium production and matrix mineralization were found to be reduced upon osteogenic differentiation in osteoprogenitors from remodeling compared with non-remodeling subchondral bone tissues within the same joint. These findings highlight the involvement of osteoprogenitor recruitment and potential perturbation of their osteoblastic differentiation and maturation potential in subchondral bone sclerosis.

The aim of our study was to investigate the osteogenic differentiation potential in vitro and in vivo of osteoprogenitor cells isolated from marrow tissues of non-sclerotic and sclerotic subchondral bone regions in human primary knee and hip OA. Osteoprogenitor cells displayed overall impaired in vitro matrix mineralization despite efficient induction of ALP activity at clonal and polyclonal levels. Ectopic bone formation assays in vivo revealed a specific reduction of osteoblast maturation in osteoprogenitors from sclerotic subchondral bone.

## 2. Results

### 2.1. Assessment of Clonogenic and Osteogenic Potential of Osteoprogenitors from Osteoarthritic Subchondral Bone

**Figure 1:**
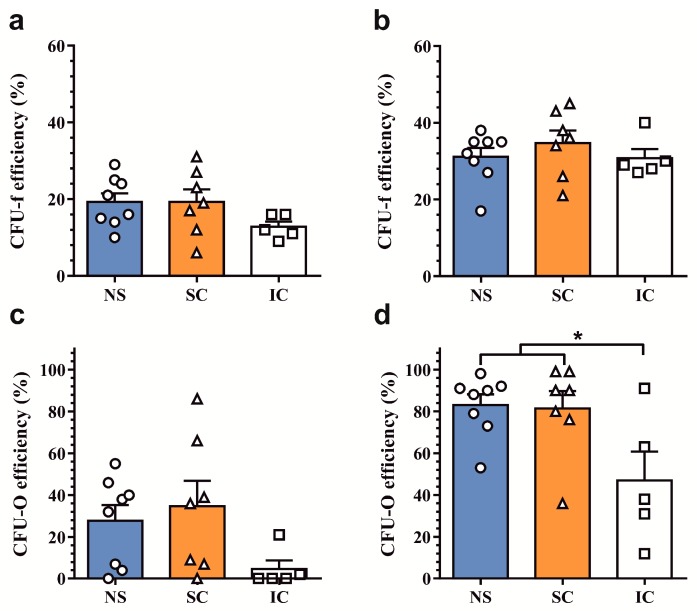
Assessment of clonogenic and osteogenic potential of osteoprogenitors from non-sclerotic (NS) and sclerotic (SC) osteoarthritic subchondral bone and non-osteoarthritic controls from iliac crest (IC). Efficiency of fibroblastic colony forming unit (CFU-f) formation in (**a**) complete and (**b**) osteogenic medium. Osteogenic potential (CFU-O), assessed as the percentage of alkaline phosphatase (ALP)-positive CFU-f colonies in (**c**) complete and (**d**) osteogenic medium. Data are presented as scatter dotplot with means + SEM, * *p* < 0.05 by one-way ANOVA.

First, we determined the clonogenicity of minimally-expanded osteoprogenitor cells isolated from non-sclerotic and sclerotic regions of osteoarthritic knee (*n* = 5) and hip (*n* = 3) joints. Osteoprogenitor cells from iliac crest biopsies served as non-OA controls (*n* = 5). The average colony-forming efficiency of first-passage osteoprogenitor cells in complete medium was 17.6 ± 1.6% and there were no differences among the groups (Figure 1a). Colony-forming efficiency was significantly increased in osteogenic medium (32.3 ± 1.6%, *p* < 0.0001), yet similar among the groups (Figure 1b). Approximately one-quarter of the colonies were ALP positive (24.4 ± 5.7%) in the absence of osteogenesis-inducing factors (Figure 1c). Treatment with an osteogenic medium strongly increased the number of ALP-positive colonies (73.5 ± 5.8%, *p* < 0.0001). While there was no difference in osteogenic potential between non-sclerotic and sclerotic regions, the relative number of ALP-positive colonies was greater in osteoprogenitors from osteoarthritic subchondral bone (82.3 ± 4.5%) than non-OA controls from the iliac crest (47.0 ± 13.7%, *p* < 0.05) (Figure 1d). 

These results suggest that the clonogenicity and osteogenic potential of native subchondral bone osteoprogenitor cells are not altered between non-sclerotic and sclerotic regions in the osteoarthritic joint. 

### 2.2. In Vitro Osteogenic Differentiation Properties of Polyclonal Osteoprogenitor Cell Populations

**Figure 2:**
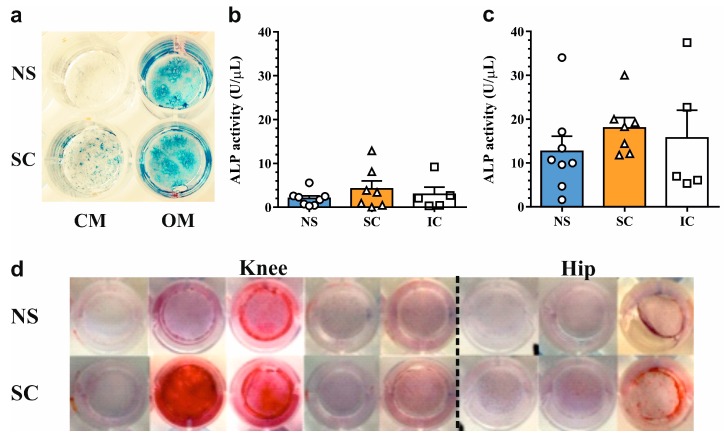
Osteogenic differentiation of osteoprogenitors from osteoarthritic subchondral bone. (**a**) Visualization of ALP activity in osteoprogenitors cultured in complete and osteogenic medium for three weeks. Quantification of ALP enzymatic activity in (**b**) complete and (**c**) osteogenic medium. Data are presented as scatter dotplot with means + SEM. (**d**) Evaluation of matrix mineralization (red) in osteoprogenitors from osteoarthritic knee and hip joints by alizarin red staining. NS: non-sclerotic, SC: sclerotic, IC: iliac crest, CM: complete medium, OM: osteogenic medium.

Next, we sought to investigate the phenotypical characteristics of osteoprogenitor cells upon osteogenic differentiation in vitro. Second-passage cells underwent glucocorticoid-induced osteogenesis for three weeks and ALP expression and matrix mineralization was determined using histochemical staining. Culture in osteogenic media led to an increase in ALP staining intensity in both non-sclerotic and sclerotic osteoprogenitor cells (Figure 2a). These findings were corroborated by quantitative assessment of ALP levels, which demonstrated a 6.3-, 4.3-, and 5.3-fold upregulation (*p* < 0.01) of ALP activity in non-sclerotic, sclerotic, and non-OA control osteoprogenitor cells, respectively. There were no differences in ALP levels among the groups under basal and osteogenic culture conditions (Figure 2b,c). Despite efficient induction of ALP activity, polyclonal osteoprogenitor cells from osteoarthritic joints displayed blunted in vitro matrix mineralization. Proper matrix mineralization was observed in only 2/16 samples (Figure 2d). These findings suggest that the mineralization capacity of native subchondral bone osteoprogenitors might be impaired in osteoarthritic joints.

### 2.3. In Vivo Osteogenic Differentiation Properties of Polyclonal Osteoprogenitor Cell Populations

**Figure 3:**
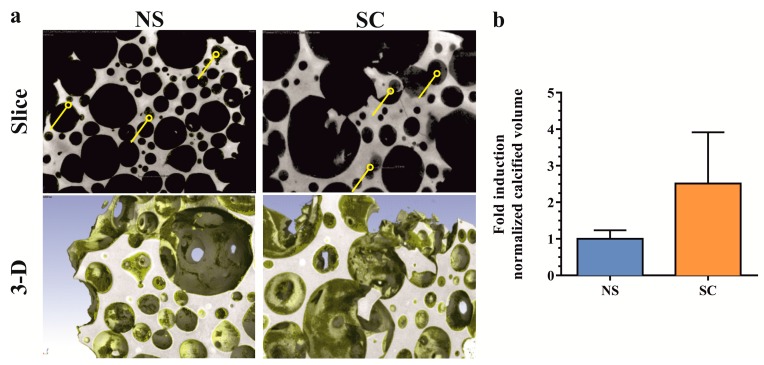
Quantification of de novo calcified tissue by micro-computed tomography. (**a**) Evidence of de novo calcified tissue formed in scaffold pores (arrowheads, upper panels). Three-dimensional visualization of ceramic (grey) and de novo calcified tissue (yellow) in explanted scaffold (lower panels). (**b**) Quantification of calcified tissue volume normalized by scaffold pore volume. Values are expressed as fold induction over non-sclerotic samples (means + SEM, *n* = 3 donors, duplicate implantations). NS: non-sclerotic, SC: sclerotic.

The in vivo osteogenic differentiation properties of osteoprogenitor cells from non-sclerotic and sclerotic regions (*n* = 3, knee joints) were evaluated in a subcutaneous implantation model of ectopic bone formation. Porous ceramic scaffolds seeded with low passage progenitor cells were explanted eight weeks after implantation. Qualitative micro-computed tomography analysis indicated the formation of calcified tissue predominantly inside smaller scaffold pores for both non-sclerotic and sclerotic osteoprogenitor cells (Figure 3a). Intradonor comparison revealed no significant differences in calcified tissue volume between sclerotic and non-sclerotic osteoprogenitor cells (Figure 3b). These results show that despite the impaired matrix mineralization in vitro, the native subchondral bone osteoprogenitors from osteoarthritic joints were able to form calcified tissue in vivo. 

### 2.4. Histological Evaluation of De Novo Calcified Tissues

**Figure 4:**
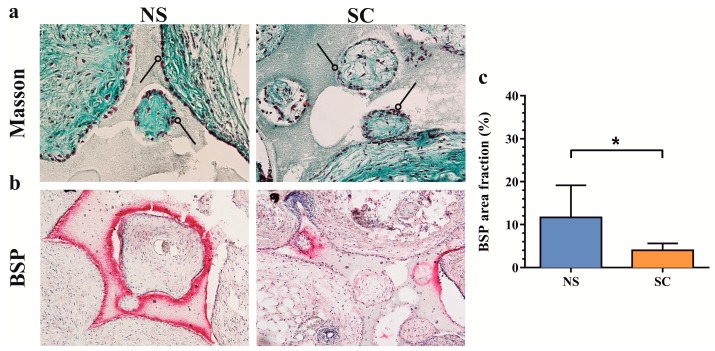
Histological characterization of de novo calcified tissues formed by osteoprogenitors from osteoarthritic subchondral bone. (**a**) Masson’s trichrome staining reveals osteoblasts (arrowheads, red nuclei) lining osteoid tissue (dense green stain). (**b**) Expression of bone sialoprotein (BSP, red) overlapping with osteoblast-like cells. (**c**) Quantification of the bone sialoprotein area normalized for scaffold pore volume (means + SEM, *n* = 3 donors, duplicate implantations). Magnification: 20×, NS: non-sclerotic, SC: sclerotic. * *p* < 0.05 by paired *t*-test.

Masson’s trichrome staining of histological tissue sections provided evidence of bone formation in the smaller scaffold pores and at the edges of larger pores with the presence of bone-lining osteoblasts (Figure 4a). The presence of active osteoblasts was confirmed by immunohistochemical staining for bone sialoprotein (BSP), a marker of late osteoblastic differentiation and positive regulator of matrix mineralization [16] (Figure 4b). Quantitative assessment of BSP staining demonstrated less expression in sclerotic compared with non-sclerotic subchondral bone osteoprogenitors. These results suggest that the native osteoprogenitors from the sclerotic subchondral bone were impaired in osteoblast maturation, but not differentiation.

## 3. Discussion

Clonogenic, multipotential progenitor cells from different tissue sources have been the topic of exhaustive studies due to their potential to regenerate skeletal tissues in a variety of disorders, including OA [17]. The contribution of joint-resident progenitor cells to the pathophysiology in musculoskeletal disorders has received only scarce attention thus far. Given that subchondral bone sclerosis is a prominent radiological feature and is considered as a promising treatment target in OA [18], we investigated whether perturbations in subchondral bone-resident osteoprogenitor cells might be involved in the formation of hypomineralized bone tissue that is associated with sclerosis. Our findings showed high osteogenic potential at a clonal level and overall impaired matrix mineralization in polyclonal cultures of osteoprogenitors from knee and hip OA joints. In vivo bone formation assays revealed a specific reduction of osteoblast maturation, evidenced by decreased BSP expression, in osteoprogenitors isolated from regions with subchondral bone sclerosis.

A marked characteristic of OA subchondral bone-resident progenitor cells was the approximately two-fold higher osteogenic potential at the clonal level compared with iliac crest biopsies of non-OA controls. Differences in proliferative capacity and osteogenic differentiation potential of progenitor cells from different skeletal sites in humans have been described previously [19]. Progenitors from non-loaded bone tissue (iliac crest) displayed lower matrix mineralization and ALP activity in vitro than cells from loaded bone tissue (orofacial). Moreover, in vivo ectopic bone formation by progenitor cells from different skeletal sites gave rise to a strikingly different histological appearance of de novo bone tissues. Therefore, it has been suggested that site-specific functional demands are molecularly imprinted in the progenitor cells residing in skeletal tissues. Furthermore, lineage tracing experiments in mice have identified distinct skeletal stem cell populations that differ with respect to clonogenicity, differentiation potential and contribution to postnatal skeletal development, and fracture repair [20]. In particular, progenitor cells expressing Gremlin-1 were found to exhibit high clonogenicity and were able to generate and maintain cartilage and bone, but not fat tissues. Differential expression of Gremlin-1 was also demonstrated in progenitor cells isolated from iliac crest biopsies or facet joint OA specimens [21]. In addition, spontaneous adipogenesis during osteogenic differentiation was typically much more frequent in progenitors from the iliac crest than subchondral trabecular bone from facet joint OA specimens. Reduced adipogenic potential and increased osteogenic potential have also been described in progenitors from the subchondral bone tissue of knee OA compared with bone marrow aspirates [22]. Colony formation assays under control and osteogenic conditions combined with immunohistochemical staining for Gremlin-1 would be insightful to explore the relationship between clonal phenotype and osteogenic potential and differences between distinct skeletal sites.

Despite their high osteogenic potential at a clonal level, osteogenic differentiation of polyclonal cultures of non-sclerotic and sclerotic subchondral bone-resident progenitors revealed severely blunted matrix mineralization in vitro. Similarly, hypomineralization has been described in multipotential stromal cells from bone marrow lesions in hip OA [15] and non-union bone tissue in temporomandibular joint ankylosis [23]. Bone marrow lesions adjacent to the subchondral bone plate in knee OA displayed increased bone mass but reduced tissue mineral density [8]. Considering the pivotal role of progenitor/multipotential stromal cells in homeostasis and repair of bone tissue, we hypothesize that molecular alterations are a cause rather than a consequence of aberrant bone formation. The molecular mechanisms underlying impaired matrix mineralization in osteoprogenitor cells remain elusive. Elevated endogenous TGF-β expression leading to aberrant type I collagen metabolism has been identified in hypomineralization of sclerotic subchondral bone osteoblasts [13]. Gene expression arrays have shown slightly increased TGF-β1 and reduced type I collagen A2 mRNA levels in progenitor cells from lesional compared with non-lesional bone marrow tissues in hip OA [15]. In contrast, progenitor cells from explant culture of subchondral bone tissues from patients with end-stage knee OA showed lower TGF-β1 expression and higher matrix mineralization than donor-matched tibial shaft marrow aspirates [22]. High concentrations of TGF-β1 have been found in subchondral bone marrow tissues in human and experimental OA and transgenic overexpression of active TGF-β1 in subchondral bone-resident osteoblasts induced an OA-like phenotype [5]. The prominent role of TGF-β signaling in recruitment, differentiation, maturation, and mineralization of osteoprogenitor cells warrants further exploration as a potential therapeutic target in human OA. Our findings showed that blunted in vitro matrix mineralization did not depend on the presence of subchondral bone sclerosis in primary knee and hip OA. However, the effects of perturbations in progenitor cells and their osteogenic differentiation on the material properties of subchondral bone are expected to be much larger in sclerotic than non-sclerotic regions, taking into account that the rate of bone formation and turnover is strongly elevated prior [24]. 

The molecular cues for the induction of osteogenic differentiation are different between in vitro two-dimensional cell cultures and a scaffold-based ectopic implantation model. Indeed, despite the lack of in vitro matrix mineralization, progenitor cells from non-sclerotic and sclerotic regions were both able to form de novo calcified tissue upon ectopic implantation. While there were no significant differences in the volume of calcified tissue formed, BSP expression was clearly reduced in osteoprogenitors from sclerotic subchondral bone. Studies in knockout mice have convincingly demonstrated that BSP, a member of the small integrin-binding ligand N-linked glycoproteins (SIBLING) extracellular matrix protein family, is a master regulator of mineralization in vitro and in vivo [25]. It has not been investigated whether BSP-null mice are prone to develop OA but primary cortical bone formation and mineralization are clearly altered [26]. Interestingly, mice lacking expression of dentin matrix protein 1, another SIBLING protein, developed an OA-like phenotype displaying increased non-calcified osteoid tissue formation in subchondral bone [27]. In human progenitor cells from the iliac crest and jaw periosteum, effective osteoinductive properties in vivo were described to correlate with higher BSP expression in vitro [28]. Furthermore, it has been demonstrated that BSP serum levels are inversely correlated with the presence of subchondral bone sclerosis in patients with knee OA [29]. Together, these findings suggest that reduced BSP expression is a prominent feature of subchondral bone sclerosis in OA and its expression might be specifically altered in progenitor cells.

The present study analyzed only a small number of OA specimens, a subset of those under in vivo conditions, and did not include healthy controls from knee and hip joints. Due to these limitations, the findings cannot be extrapolated to the OA patient population in general. In light of the recent definition of different clinical OA phenotypes with a varying involvement of the subchondral bone tissue, the novel pathomechanism uncovered in this study most likely plays a role in the bone phenotype rather than in inflammatory, metabolic, or osteoporotic phenotypes [3,30,31]. A second limitation comprises the variation in cell isolation and expansion protocols and the nomenclature between the present and previously published studies [15,21,22]. Consensus rules for defining true stem cells have been laid down for bone marrow aspirates [32], a common source for cell-based regenerative therapies, but not fatty marrow of trabecular bone tissues. Regardless of the cell isolation protocol, alterations in the osteogenic differentiation properties and in vitro matrix mineralization seem to be a common phenomenon in joint pathology. Further investigations are required to shed light on the relationship between subchondral bone sclerosis, bone marrow lesions, and subchondral bone-resident progenitor cell properties in different OA phenotypes. 

In conclusion, we have demonstrated that progenitor cells from OA subchondral bone display high osteogenic potential at a clonal level and blunted in vitro matrix mineralization at a polyclonal level. Progenitors from sclerotic subchondral bone have shown lower BSP expression during in vivo bone formation, providing a plausible pathomechanism for subchondral bone sclerosis in OA. Due to their pivotal role in generating and maintaining pathological bone tissue changes, subchondral bone progenitor cells may be a novel OA treatment target.

## 4. Materials and Methods

### 4.1. Patients

Patients with primary knee (*n* = 5) or hip (*n* = 3) osteoarthritis (average age 69.5 ± 4.1; range 52–87, five female) scheduled to undergo total joint arthroplasty were recruited into the study. Exclusion criteria included a history of inflammatory arthritis, metastatic cancer, or bone-related disorders. As controls, iliac crest trabecular bone grafts were obtained from age-matched patients (*n* = 5) undergoing spine fusion surgery for lumbar spinal stenosis (average age 66.4 ± 2.5; range 59–74; four female). Written informed consent was obtained from all patients. The study protocol had been reviewed and approved by the local ethics committee (No. 147/12, 29 May 2012).

### 4.2. Enzymatic Release and Culture of Osteoprogenitor Cells

Freshly obtained tibial plateaus (knee), femoral heads (hip), and iliac crest bone were rinsed with sterile phosphate-buffered saline (PBS). Non-sclerotic and sclerotic regions within the same joint were identified by visual inspection; non-sclerotic regions were covered by cartilage without surface irregularities, whereas sclerotic regions were denuded or covered by severely debrided cartilage lesions. Osteochondral slabs (2 × 2 cm^2^) were prepared from both regions using a bone saw and cartilage was removed with a scalpel. Bone slabs were sagittally cut in ~1 mm thick chips with a scalpel. Osteoprogenitor cells/bone marrow stromal cells were released from subchondral bone marrow tissues by collagenase digestion as described previously [21,33]. Fragments (±1.0 g wet weight) were subjected to enzymatic digestion in 10 mL DMEM supplemented with 0.6 mg/mL clostridial collagenase IA (Sigma-Aldrich, Buchs, Switzerland). Samples were digested for 3 h at 37 °C and vortexed every 20 min. Bone fragments were thoroughly rinsed in sterile PBS and solutions added to the collected digestion medium. Digestion medium was consecutively passed through 70 and 40 µm cell strainers (SLG, Hartmannsdorf, Germany), centrifuged at 350× *g* and resuspended in 5 mL complete medium (αMEM supplemented with antibiotics, 10% fetal bovine serum, 10 mM HEPES, and 4 mM glutamine (Sigma-Aldrich). The single cell suspension was seeded in a 6-well plate and expanded to subconfluency with medium changes twice per week. Osteoprogenitors/bone marrow stromal cells were selected on the basis of adhesion and proliferation to the plastic substrate. Cells (passage 1) were harvested by trypsinization and further expanded to subconfluency in 75 cm^2^ flasks.

### 4.3. Colony-Forming Unit Assays

For colony forming unit (CFU) assays, subconfluent first passage cells were harvested by trypsinization and seeded in triplicate in 6-well plates at a density of 10 cells/cm^2^ (100 per well) in complete medium containing 2.5 ng/mL recombinant human fibroblast growth factor-2 (FGF-2, R&D Systems, Abingdon, UK) or osteogenic differentiation medium (complete medium supplemented with 10^−7^ M dexamethasone, 50 µM l-ascorbic acid-2-phosphate, and 10 mM sodium beta-glycerophosphate pentahydrate (Sigma-Aldrich)). After culture for two weeks under bi-daily medium changes, cells were fixed in formalin for 15 min at room temperature. Osteogenic colonies (CFU-O) were identified and enumerated by staining for alkaline phosphatase (ALP) activity through incubation in 0.1 M Tris-HCl pH 8.2 containing 0.1 mg/mL naphthol AS-MX phosphate and 0.3 mg/mL fast blue BB salt (Sigma-Aldrich) at 37 °C for 1 h. Total fibroblastic colonies (CFU-f) were counted after crystal violet staining. Only colonies containing more than 50 cells were counted. The osteogenic potential was calculated as the ratio CFU-O/CFU-f in each well.

### 4.4. Osteogenic Differentiation Assays

Subconfluent passage 1 cells were harvested by trypsinization and seeded at approximately 80% confluency in 24-well plates. Confluent cells were cultured in complete medium with 2.5 ng/mL FGF-2 or osteogenic medium for three weeks with medium changes twice per week. Cells were rinsed in PBS and either left untreated for quantitative ALP activity assay or fixed in formalin for 15 min at room temperature for alizarin red and ALP staining.

### 4.5. Quantitative Alkaline Phosphatase Activity Assay

ALP activity was determined in triplicate. Cells were lysed in 0.01% sodium dodecyl sulfate and ALP activity in lysates was determined by spectrophotometric measurement at 405 nm of the hydrolysis of *p*-nitrophenyl phosphate in alkaline buffer for 15 min at 37 °C. Results are reported as units/μL (1 unit hydrolyzes 1 mM *p*-nitrophenyl phosphate per minute at pH 9.8 at 37 °C).

### 4.6. Alizarin Red S Staining

Fixed duplicate wells were incubated in alizarin red S staining solution (2% alizarin red S in ddH_2_O-NaOH pH 4.2) for 10 min at room temperature. Wells were subsequently rinsed with two volumes ddH_2_O and one volume 70% isopropanol and photographed.

### 4.7. Subcutaneous Implantation Model of Ectopic Bone Formation

Osteoprogenitor cells from non-sclerotic and sclerotic subchondral bone (*n* = 6, three donors, knee osteoarthritis) were expanded through passage 3–5 in complete medium supplemented with 2.5 ng/mL FGF-2. One million cells were seeded on porous hydroxyapatite cylinders (Engipore, Finceramica-Faeza, Faeza, Italy) and implanted into subcutaneous pouches of nude mice (CD-1 nude/nude, Charles River Laboratories, Ashland, OH, USA). Four pouches were implanted per mouse with duplicate grafts per donor. After eight weeks, mice were euthanized and grafts were explanted and immediately fixed in formalin for two days.

### 4.8. Micro-Computed Tomography Scanning and Analysis

Explanted grafts were transferred to PBS and scanned in a nano-scale resolution X-ray tomography system (phoenix|X-ray, GE Sensing & Inspection Technologies GmbH, Wunstorf, Germany). Scanning settings were: 70 kV, 260 μA, 360° scan, 0.5 mm aluminum filter. Three-dimensional reconstructions were performed using the manufacturer’s software (phoenix datos|x 2.0.1 RTM, GE Sensing & Inspection Technologies GmbH) and quantification of de novo calcified tissue was determined with VGStudio Max (Version 2.2, Volume Graphics, Heidelberg, Germany).

### 4.9. Histological Analysis

Samples were completely decalcified in 15% EDTA pH 7.0 in ddH_2_O and embedded in paraffin. Histological tissue sections (7 μm) were stained with Masson’s trichrome (Réactifs RAL, Martillac, France) as previously described [34,35]. Expression of bone sialoprotein (BSP) was determined using immunohistochemistry. Binding of BSP primary antibody (ab52128, Abcam, Cambridge, UK) was visualized using Vectastain Fast Red kit (Vector Laboratories, Glostrup, Denmark) according to the manufacturer’s instructions. Images were taken with a BX61 microscope (Olympus, Tokyo, Japan). Quantification of BSP staining area per scaffold area was performed using ImageJ (National Institutes of Health, Bethesda, MD, USA).

### 4.10. Statistical Analysis

Statistical analyses were performed using GraphPad Prism (v6.2, GraphPad Software Inc., La Jolla, CA USA). Data are reported as the mean ± standard error of the mean (SEM). Significant differences were calculated using paired *t*-test or one-way analysis of variance (ANOVA). *p*-Values less than 0.05 were considered significant. 

## 5. Conclusions

In conclusion, we have demonstrated that progenitor cells from OA subchondral bone display high osteogenic potential at a clonal level and blunted in vitro matrix mineralization at a polyclonal level. Progenitors from sclerotic subchondral bone have shown lower BSP expression during in vivo bone formation, providing a plausible pathomechanism for subchondral bone sclerosis in OA. Due to their pivotal role in generating and maintaining pathological bone tissue changes, subchondral bone progenitor cells may be a novel OA treatment target.

## Abbreviations

OA	osteoarthritis
ALP	alkaline phosphatase
BSP	bone sialoprotein
CFU	colony forming unit
